# 

**Published:** 1909-04-24

**Authors:** 


### BOOKS RECEIVED.

John Wright and Sons, Ltd.

"Health, Morals and Longevity." By George Gresswell, M.A., L.R.C.P., and Albert Gresswell, M.A., M.D.

"Medical Annual," 1909.

Adlard and Son.

"Medical Reports of the Central London Throat and Ear Hospital."

H. K. Lewis.

"The Causation of Sex." By E. Rumley Dawson, L.R.C.P., M.R.C.S.

Ward, Lock, and Co.

"Mrs. Beeton's Cookery Book."

J. W. Arrowsmith, Bristol.

"Dromina." By John Ayscough.

John Bale, Sons and Danielsson, Ltd.

"Notes and Thoughts from Practice." By W. J. Tyson, M.D., F.R.C.P., F.R.C.S.

"A History of the Reading Pathological Society." By J. B. Hurry, M.A., M.D.

"Tuberculin in Diagnosis and Treatment." By Dr. Bandelier and Dr. Roepke.

### ANSWERS TO CORRESPONDENTS.

Nemos.—In reply to your question, Sir Aston Webb remodelled the Stafford Hospital and designed a hospital at Burton. We do not know of any other hospital work with which he has been concerned.

The rate of annual subscriptions to The Hospital, either direct from the Office or through agents, is:—

For the United Kingdom ... ... ... 15s. a year
To the Colonies and Abroad ... ... 17s. a year
inclusive of postage in each case.

